# Supplementary material for: High-Fat Diet-Induced Obesity Increases Brain Mitochondrial Complex I and Lipoxidation-Derived Protein Damage
Source: Antioxidants (Basel). 2024 Jan 26;13(2):161. doi: 10.3390/antiox13020161 (PMC10886272; doi:10.3390/antiox13020161)

Figure S1: Western Blots raw data.

Figure 2A
Anti-NDUFV2
SAB2107279,Sigma

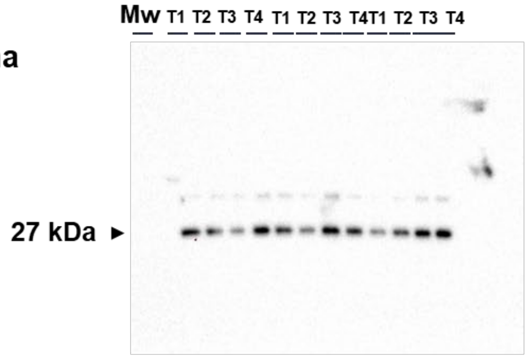

Figure 2A
Anti-NDUFS3
459130, Invitrogen

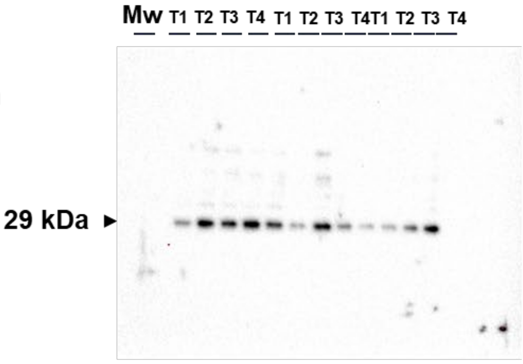

Figure 2A
Anti-NDUFS4
96549, Abcam

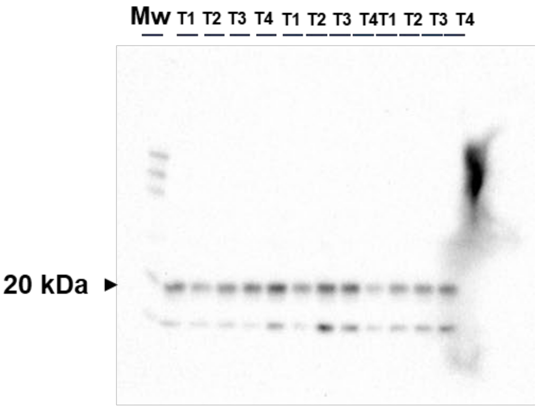

Figure 2A
Anti-NDUFA9
459100, Life Tech

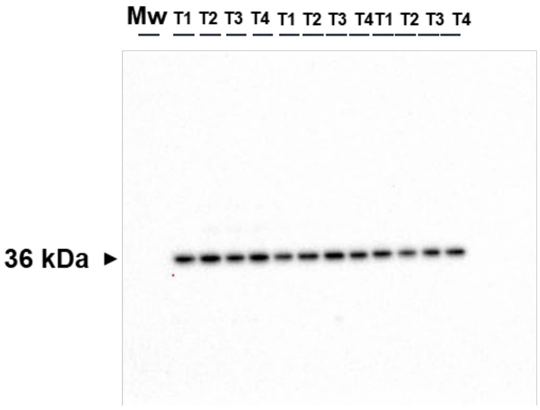

Figure 2A
Anti-VDAC/Porin
Ab15898, Abcam

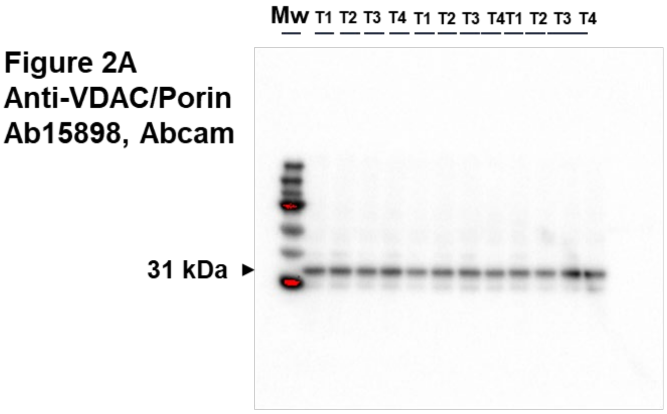

Figure 2A
Coomassie Blue

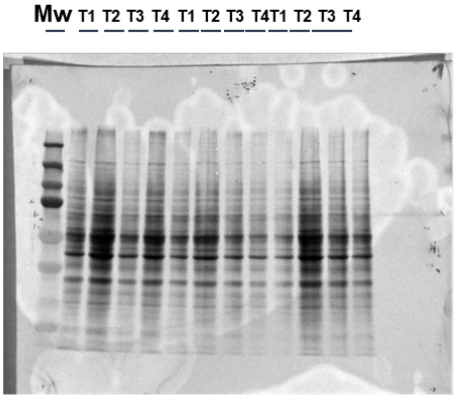

Figure 2B
Anti-Flavoprotein (Complex II)
459200, Invitrogen

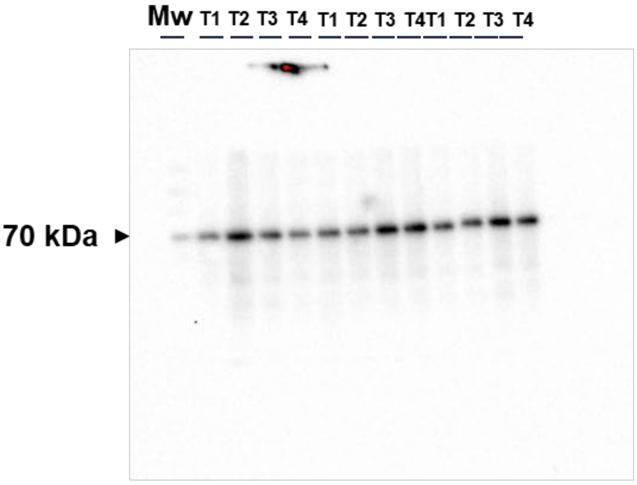

Figure 2B
Anti-VDAC/Porin
Ab15898, Abcam

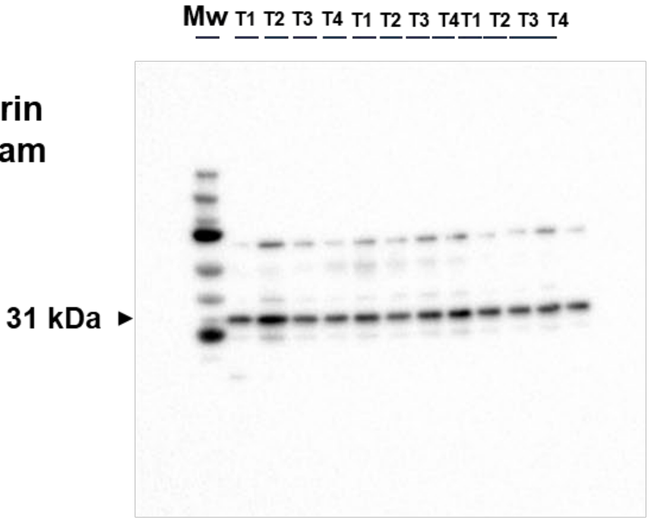

Figure 2B
Coomassie Blue

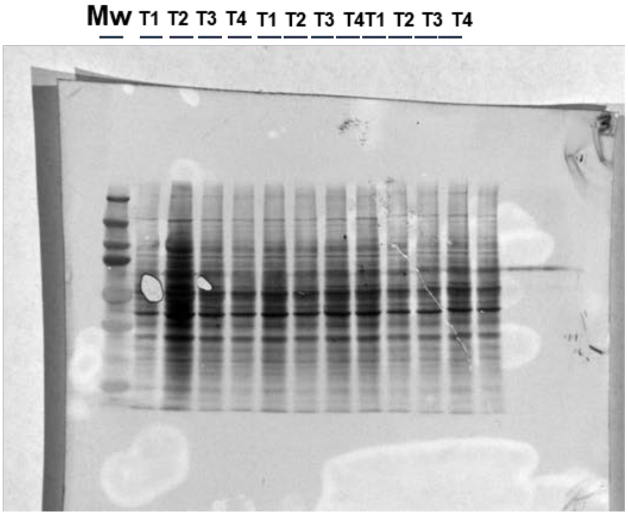

Figure 2C
Anti-CORE2 (Complex III)
459220, Invitrogen

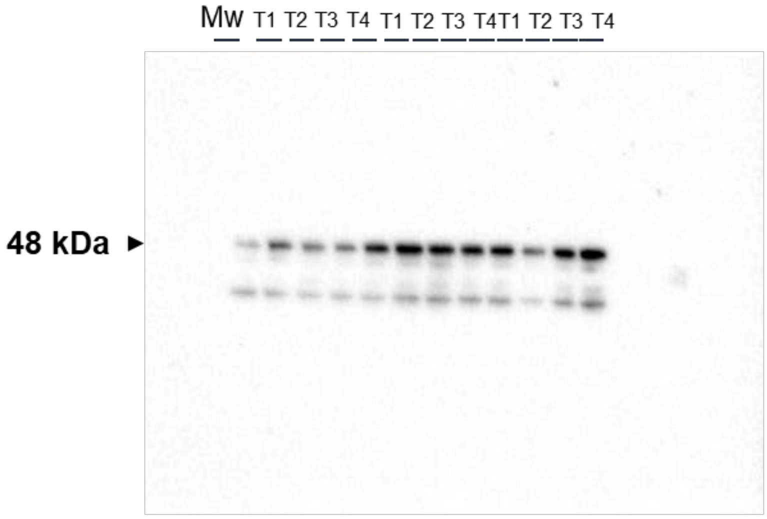

Figure 2C
Anti-VDAC/Porin
Ab15898, Abcam

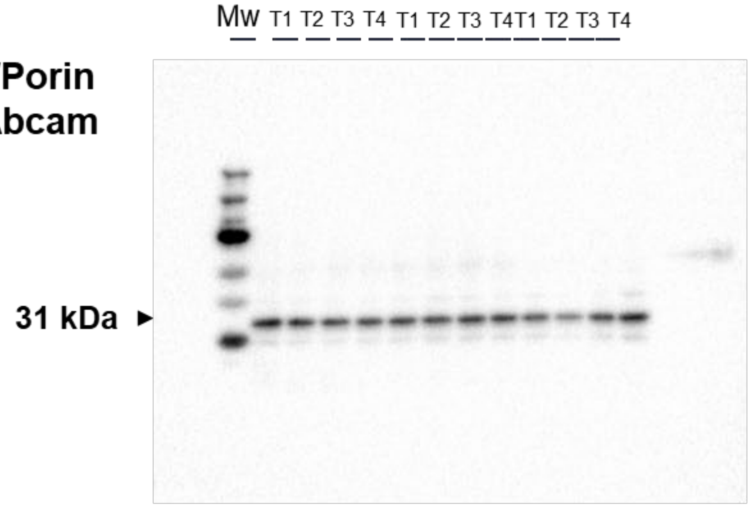

Figure 2C
Coomassie Blue

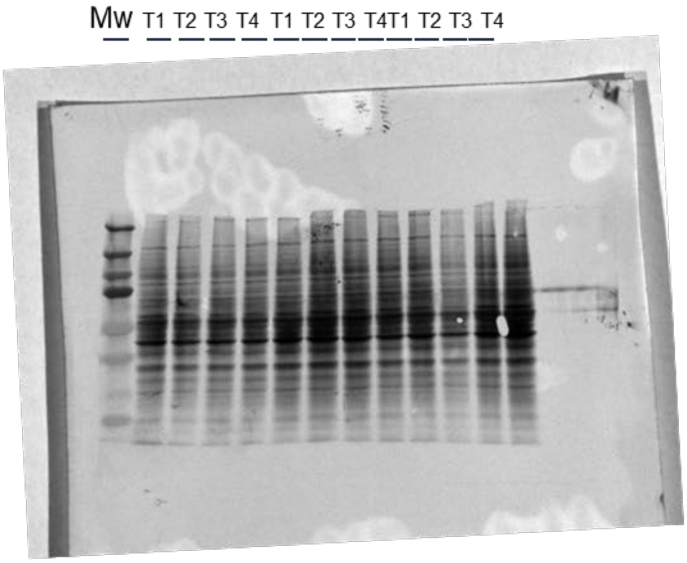

Figure 2D
Anti-COXI subunit
(Complex IV)
59600,Invitrogen

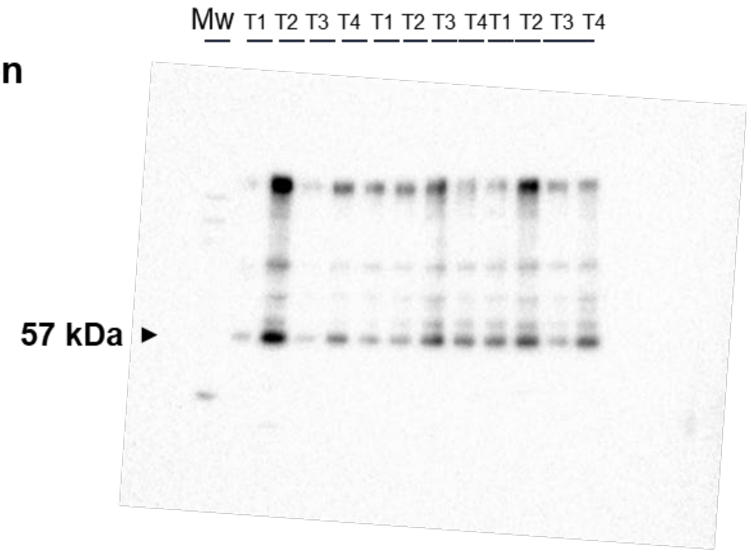

Figure 2D
Anti-VDAC/Porin
Ab15898, Abcam

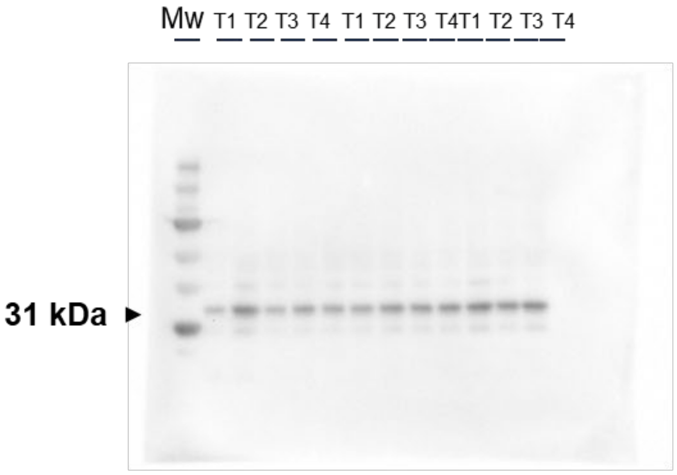

Figure 2D
Coomassie Blue

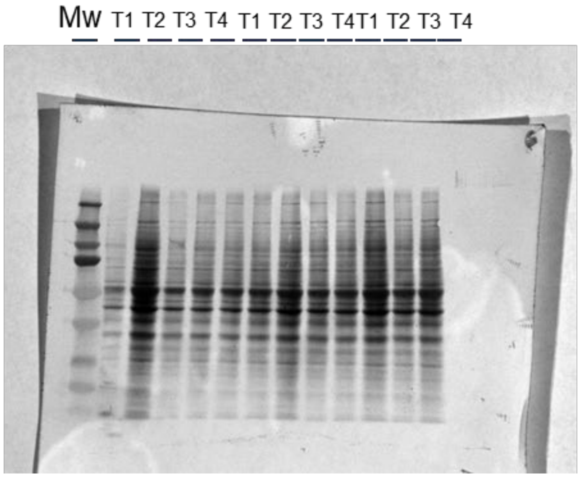

Supplement: Supplementary file 1 [file antioxidants-13-00161-s001.zip › antioxidants-2756486-supplementary.pdf]
